# Impact of Dietary Macronutrient Intake during Early and Late Gestation on Offspring Body Composition at Birth, 1, 3, and 5 Years of Age

**DOI:** 10.3390/nu10050579

**Published:** 2018-05-08

**Authors:** Christina Brei, Lynne Stecher, Dorothy Marie Meyer, Veronika Young, Daniela Much, Stefanie Brunner, Hans Hauner

**Affiliations:** 1 From the Else Kröner-Fresenius-Center for Nutritional Medicine, Klinikum rechts der Isar, Technical University of Munich, 80992 Munich, Germany; christina.brei@tum.de (C.B.); lynne.stecher@tum.de (L.S.); dora.meyer@tum.de (D.M.M.); veronikayoung3@gmail.com (V.Y.); d.much@gmx.de (D.M.); stefanie_brunner@gmx.de (S.B.); 2 ZIEL–Institute for Food and Health, Nutritional Medicine Unit, Technical University of Munich, 85354 Freising, Germany

**Keywords:** body composition, child adiposity, dietary intake, pregnancy

## Abstract

Dietary intake during pregnancy as a possible modifiable risk factor for childhood obesity is poorly explored. In a prospective observational study, two multivariable regression models were therefore used to associate maternal diet at 15 and 32 weeks’ gestation with offsprings’ body composition and fat distribution at birth, 1, 3, and 5 years. Mean energy intake was 2157 ± 375 kcal (*n* = 186) in early and 2208 ± 460 kcal (*n* = 167) in late gestation. The partition model showed mostly no significant associations between maternal diet in early pregnancy and offspring body composition. In late pregnancy, higher fat intake was negatively associated with clinical outcomes at birth, 1, and 5 years. Protein intake was negatively associated with BMI *z* score (zBMI) at 3 and 5 years. A 10 g increase in fiber was associated with an increase of 3.50 mm^2^ abdominal subcutaneous fat at 1, 172.49 g fat mass at 3, and 0.23 zBMI at 5 years. Results were largely comparable in the substitution model. An incremental increase in fat and protein at the expense of carbohydrates in late but not early pregnancy may be associated with lower fat mass up to 5 years. Findings require confirmation by additional prospective studies.

## 1. Introduction

Overweight and obesity have become growing health problems with alarmingly high prevalence rates found not only in adulthood, but also in childhood and adolescence [1]. Further, data clearly shows that children who are overweight/obese have an increased risk of being overweight/obese in adulthood compared to their normal-weight peers [2,3]. To tackle this epidemic, recent research activities are focusing on perinatal strategies to prevent obesity in childhood.

The “fetal programming hypothesis” suggests that circumstances occurring during pre- and postnatal periods affect health outcomes of the offspring later in life [4,5]. An unbalanced, suboptimal maternal nutrition, i.e., maternal under- or overnutrition, can affect offsprings’ later metabolic health risk and increase susceptibility to obesity in adulthood [6], as originally suggested by follow-up studies analyzing the long-term outcomes of the Dutch hunger winter (1944/45) [7,8].

Over the past decade, there has been growing interest on the impact of maternal diet on offspring’s body weight and body composition under non-extreme conditions. Further, it remains unclear if there is a particular time window during gestation in which the fetus is particularly sensitive to alterations in the maternal diet. To date, studies have shown inconsistent results, in part due to different approaches in dietary assessment methods, different time-points of data collection, body composition measuring techniques, statistical analyses, and methodological limitations [9,10]. Further, few studies have addressed alterations in fat distribution [11,12], a key predictor of obesity-related complications [13,14]. Most studies have only examined outcomes at birth without considering the potential long-term effects of gestational nutrition.

A secondary analysis of data from a randomized controlled trial [15] has therefore set out to answer the following research questions: First, is there a critical time period during pregnancy in which alterations to maternal macronutrient intake has a greater influence on offspring adiposity and its distribution in newborns? Second, do any of the observed effects persist during infancy and early childhood? For this purpose, the maternal intake of selected macronutrients during early and late gestation was assessed for its association with direct and indirect offspring body composition parameters, at birth, 1, 3, and 5 years of age within the same group of mother-child pairs.

## 2. Materials and Methods

### 2.1. Study Design and Subjects

Data came from an open-label, monocenter, randomized controlled trial with a two-arm parallel group design. The primary objective of the trial was to investigate whether an *n*-3 long-chain polyunsaturated fatty acid (PUFA) supplementation during pregnancy and lactation affects early human adipose tissue development in the offspring [15]. In brief, 208 pregnant healthy women were randomly assigned to one of two groups. Women in the intervention group received 1.2 g *n*-3 long-chain PUFAs as a supplement between the 15th week of gestation and 4 months postpartum and dietary counselling to lower arachidonic acid intake. In contrast, women assigned to the control group obtained standard counselling on a healthy diet during pregnancy at 15 weeks’ and 32 weeks’ gestation. As no significant evidence of an effect in regard to fat mass and its distribution was found in the adjusted models from birth up to 5 years of life [16,17], data were pooled for the present analysis. Here, we focused on mother-child pairs for whom maternal dietary data and body composition data in the offspring were available. All participants gave their informed consent for inclusion before they participated in the study. The study was conducted in accordance with the Declaration of Helsinki, and the protocol was approved by the Ethics Committee of The Technical University Munich (ClinicalTrials.gov, number ID NCT00362089, http://clinicaltrials.gov/ct2/show/NCT00362089). 

### 2.2. Dietary Record Collection and Analysis

Women completed two 7-day dietary records; the first at early stage (at 15th week of gestation), and the second at late stage (at 32nd week of gestation) of pregnancy. Participants were advised by trained research assistants to estimate consumed food portion sizes by the provision of household measures, standard units or in grams. Dietary intake was calculated using three of seven days (one weekend day, two weekdays), excluding day 1, which were randomly chosen by a customized Microsoft Access software (Helmholtz Centre Munich, Institute of Epidemiology 2). Standardized data entry was performed by a single person (VY) using the software OptiDiet Plus (Version 5.1.2.065, GOE mbH), a program based on a German nutrient database (Bundeslebensmittelschlüssel). In accordance with Meltzer et al. [18], pregnant women with an unrealistic energy intake (mean) <4.5 megajoules (MJ) and >20 MJ (alternatively <1075 kcal and >4777 kcal) were excluded from the analysis.

### 2.3. Assessment of Body Composition

The methods for assessing body composition have been explained in detail previously [16,17]. In brief, birth weight and length were received from maternal obstetric records. At one year, weight and length were recorded in supine position using a standard infant scale (Babywaage Ultra MBSC-55; myweight) to the nearest 10 g and a measuring stick (Säuglingsmessstab seca 207; seca). At 3 and 5 years, a standard flat scale (Seca Clara 803; seca) to the nearest 100 g was used to assess weight and a stadiometer (Stadiometer seca 214; seca) to determine height to the nearest 0.5 cm in standing position. Based on a German reference group, age- and sex-standardized BMI *z* score (zBMI) were calculated [19]. Skinfold thickness measurements were performed in triplicate with a Holtain Caliper (Holtain Ltd., Crosswell, Crymych, Pembs., UK) at four body sites (biceps, triceps, subscapular, and suprailiac) at birth, 1, 3, and 5 years. Predictive skinfold regression equations were used [20] to calculate body fat mass (g). Abdominal subcutaneous and preperitoneal fat areas were measured at 6 weeks, 1, 3, and 5 years by sonography according to Brei et al. [21]. The latter is considered a proxy of visceral fat, as demonstrated in children [22] and adults [23]. Abdominal MRI measurements were performed at 5 years of age in a subgroup of children to determine volumes of subcutaneous and visceral adipose tissue as previously described [17].

### 2.4. Statistical Analysis

Continuous variables of maternal and offspring characteristics are presented as means ± SDs; discrete numbers are presented as percentages. Analyses at each time point included all mother-child pairs in our cohort for whom we had data. Comparisons of maternal and offspring characteristics and maternal energy and macronutrient components between early and late gestation were performed by using paired t-tests for continuous data. For categorical data, chi-square test was used. According to Crume et al. [9], two types of multivariable regression models were applied to explore the influence of maternal dietary intake on offsprings’ body composition parameters. The partition model calculates the estimated unit change in the respective offspring body composition parameter associated with a 100 kcal increase of the macronutrient of interest (non-isocaloric = total energy intake is not kept constant). The substitution model calculates the estimated unit change in the offspring body composition parameter associated with a 1% increase of energy of the macronutrient of interest and a respective 1% decrease of the other macronutrients (isocaloric = total energy intake is kept constant). Fiber and macronutrient intake was energy adjusted in the substitution but not the partition model. Fiber was calculated as having 2 kcal per gram. For both models, adjustments were made for sex, pre-pregnancy BMI, gestational age in days, and group allocation (intervention vs. control group). Statistical analyses were performed with PASW software (version 21.0; SPSS Inc., Armonk, NY, USA: IBM Corp) and a 2-sided *p* value of <0.05 was considered statistically significant. No correction for multiple comparisons was carried out.

## 3. Results

Of the 208 pregnant women originally included in the study, data from 186 woman-child pairs for early (15th week of gestation) and 167 woman-child pairs for late gestation (32nd week of gestation) were available at birth. A flowchart of mother-child pairs with available data at each time point is given in the Online Supplemental Material (Supplemental Figure S1). Baseline maternal and anthropometric data for newborns, as well as 1-, 3-, and 5-year-old children are provided in Table 1, separately for early and late gestation (no significant differences between the examined time points, data not given). Women included in the study were, on average, 32 years old, white and relatively well educated. Mean pre-pregnancy BMI was 22 kg/m^2^, with women predominantly categorized as normal weight (77% had a BMI between 18.5–24.9 kg/m^2^). 

### 3.1. Dietary Intake During Early and Late Gestation

Maternal dietary energy (kcal and kJ) and macronutrient intakes (grams and percentages of energy) at early and late gestation are given in Table 2. For both time points of assessment, mean daily energy intake were in the aforementioned defined range (1075–4777 kcal) with a mean energy intake of 2157 ± 375 kcal in early and 2208 ± 460 kcal in late gestation. Differences between energy and macronutrient consumption measured in early and late pregnancy were small and not statistically significant, except for saturated fatty acids (SFA) as a percentage of total energy intake, with higher values in late gestation (early gestation: 14.8 ± 3.3% SFA of energy intake; late gestation: 15.3 ± 3.7% SFA of energy intake; *p* = 0.05). 

### 3.2. Maternal Macronutrient Intake in Early Pregnancy and Offspring Body Composition

The partition model, given in Table 3, shows the association between dietary intake assessed at the 15th week of gestation on offspring body composition at birth, 1, 3, and 5 years of age. A 100 kcal increment of analyzed nutritional components was not associated with direct or indirect body composition measures at the investigated time points (for fiber 20 kcal increment), except for an association between monounsaturated fatty acids (MUFAs) and zBMI at birth, whereby an increase of 100 kcal MUFAs was related to increased zBMI by 0.28 m^3^ (95% confidence interval (CI): 0.04; 0.52; *p* = 0.021). Subgroup MRI analysis showed evidence of a negative association between fiber intake and abdominal visceral adipose tissue volume (Table 4). In this subgroup analysis, an additional non-isocaloric increment of 20 kcal of fiber (≙10 g) was associated with a decrease of visceral fat mass by 20.71 cm^3^ (95% CI: −40.64; −0.78 cm^3^; *p* = 0.042) at 5 years of age. Results for the substitution models are given in the Online Supplemental Material (Supplemental Tables S1–S3). We observednonsignificant associations with one exception: total carbohydrate intake and subcutaneous fat area at 1 year were significantly positively associated. Subgroup analysis showed consistent results, with a negative association between fiber intake and visceral fat at 5 years.

### 3.3. Maternal Macronutrient Intake in Late Pregnancy and Offspring Body Composition

Table 5 shows the results for the partition model from data collected at the 32nd week of gestation, with several isolated significant associations between dietary components and body composition measurements at birth, 1, 3, and 5 years of age. At birth, an additional intake of 100 kcal in PUFAs was associated with a 161.4 g decrease of birthweight (*p* = 0.036) and a 58.4 g decrease of fat mass (*p* = 0.028), respectively. The detected significant associations did not persist to 1 year of life. In contrast, a 100 kcal increase of total fat and SFA was associated with a significant decrease of −1.30 mm^2^ respective −2.79 mm^2^ in subcutaneous fat area, assessed by ultrasound at 1 year. A 20 kcal increment of fiber (≙10 g) was positively associated with an increase of 3.50 mm^2^ subcutaneous fat area at 1 year (95% CI: 0.36, 6.65 mm^2^; *p* = 0.029), 172.41 g increase in fat mass at 3 years (95% CI: 6.43, 338.38 g; *p* = 0.042), as well as 0.23 increase in zBMI at 5 years of age (95% CI: 0.03, 0.45; *p* = 0.023). Total protein was negatively associated with zBMI with significant results at 3 and 5 years of age. Furthermore, additional 100 kcal intake of total fat and PUFAs were negatively associated with subcutaneous fat areas at 5 years of age. No significant associations were found for subgroup analyses with abdominal MRI (Table 4).

The associations for the substitution model in late pregnancy are presented in Supplemental Table S3. Statistical analyses showed largely comparable results to the partition model. The main differences were that an isocaloric increase of 1% of PUFA (i.e., 22 kcal or 2.4 g PUFA) was not significantly associated with any measurement of body composition, while the isocaloric increase of 1% of carbohydrates was positively associated with subcutaneous fat area at 1 year and zBMI at 3 years, respectively. In addition, we observed that an isocaloric increase of 1% of sugar was positively associated with zBMI and subcutaneous fat area at 3 years, and only subcutaneous fat area at 5 years. Similar to Table 3, no significant associations were found for subgroup analyses with abdominal MRI (Supplemental Table S2).

Sensitivity analyses were performed to observe if other maternal and child variables could have influenced child outcomes. We performed linear regression in both the partition and substitution models with offspring clinical outcomes at 5 years. The confounders which were used in primary analyses, namely sex, pre-pregnancy BMI, gestational age in days, and group allocation (intervention vs. control group), with the addition of breastfeeding status at 4 months, maternal higher education attainment, maternal age, and parity, were fitted to both models. The results of the sensitivity analyses were similar to those found in our primary analyses. 

## 4. Discussion

The objective of the present study was to investigate the effect of maternal diet during early and late pregnancy on offsprings’ body composition and fat distribution at birth, 1, 3, and 5 years of life. Maternal diet assessed in the 15th week of gestation had largely no significant influence on child body composition measures, except for maternal MUFAs and zBMI at birth (partition model), and MRI-subgroup analysis, whereby fiber intake was negatively associated with abdominal visceral adipose tissue mass (both models). When dietary intake in the 32nd week of gestation was considered, fat (total fat, PUFAs and SFA) was found to be negatively related to both indirect and direct measures of body composition at birth, 1, and 5 years of age, with most significant associations found with abdominal subcutaneous fat areas assessed by ultrasound. Total protein was found to be negatively associated with zBMI at 3 and 5 years of age, while fiber was significantly positively associated with body composition parameters at 1, 3, and 5 years of age. An isocaloric increase of 1% in carbohydrates and sugar was positively associated with body composition parameters at two time points in the substitution model only.

Previous human observational studies have demonstrated positive associations between total maternal fat and/or SFA intake during pregnancy with neonatal fat mass [9,11] and offspring weight status at 5 years of age [25]. Contrary to what might be expected, this analysis suggests that higher maternal intake of not only PUFAs, but also total fat and SFAs during late pregnancy may be associated with lower body fat in offspring, assessed using different measurement methods. While we have observed these associations in late pregnancy, a recently published paper reported a negative association between fat and PUFA intake and birth weight during the first but not in the second trimester [26].

Our data further show that carbohydrate intake was not associated with body composition measures in both models at birth, while other studies have observed positive links [9,26,27]. One such study with 1040 women demonstrated that a non-isocaloric 100 kcal increase in maternal carbohydrate intake led to a 2.9 g increase in neonatal fat mass [9], while another showed that carbohydrate intake in late gestation led to greater total fat mass, including higher abdominal fat mass, in newborns [27]. Rather, our analyses point to associations with late gestational carbohydrate intake and growth and adipose measurements in infancy and early childhood. Results from the substitution model indicated that a 1% isocaloric increase of maternal total carbohydrates resulted in higher subcutaneous fat area and zBMI in 1- and 3-year-old children, which is in line with previous findings [28]. Other studies have investigated whether alterations in the quality and quantity of maternal carbohydrate intake in early pregnancy resulted in long-term body-composition changes in the offspring. In accordance with our observations, Murrin et al. found that prenatal sugar intake was associated with offspring adiposity at 5 years of age [25], while Jen et al. observed that maternal intake of sugar-containing beverages was positively associated with BMI and fat mass in children ≤6 years [29]. Positive associations have also been shown between maternal carbohydrate and sugar intake at 26–28th week of gestation with zBMI in 2-, 3-, and 4-year-old children [28].

Unexpectedly, increased intake of about 10 g fiber in late gestation showed a tendency towards an increase in different measures of adiposity at 1, 3, and 5 years of age (both models). However, no significant associations were detected for visceral fat (measured by MRI) or preperitoneal fat (measured by ultrasound), where the latter approximates visceral fat [22,23]. Conversely, fiber intake in early pregnancy was negatively associated with abdominal visceral fat at 5 years, suggesting that increased fiber intake in the first trimester may decrease abdominal fat depots in offspring in early childhood. While studies have consistently shown that a fiber rich diet is associated with a reduced risk of obesity in adults [30], research on the impact of fiber on childhood adiposity has been sparse, and the results are inconsistent [31]. Moreover, associations between maternal fiber intake and offspring adiposity are to date poorly explored and findings have been discordant. Although some studies have shown a beneficial effect of fiber rich dietary intake in pregnancy on offspring obesity risk [32], others have demonstrated that these relationships are attenuated to null when adjusting for maternal and child lifestyle factors [33]. Our results demonstrate albeit weak, but to a certain extent consistent associations between maternal macronutrient intake and fat mass and its distribution in the offspring up to the age of 5 years. It is unclear as to whether such associations are stable or may disappear during further growth, in part due to a growing influence from external factors. The prospective Avon Longitudinal Study of Parents and Children has presented the longest follow-up data so far. Maternal carbohydrate, fat and protein intake at late pregnancy (32nd week of gestation) showed no association with offsprings’ fat and lean body mass at 9 and 11 years of age [34].

While research indicates that maternal nutrient intake during pregnancy impacts offspring fat mass development [35], little is known about whether there are specific gestational periods in which the developing fetus is particularly sensitive to changes in the maternal diet. Our data suggest that maternal nutrition in late pregnancy may have the most profound influence on offspring’s body composition outcomes. These findings are in line with Renault et al., who found that carbohydrate intake in obese women at late, but not at early gestation was positively associated with fat mass at birth [27]. In contrast, Moore et al. observed that maternal dietary composition in early pregnancy, but not in the third trimester, was associated with offspring body composition outcomes [36]. A study from Sharma and colleagues found significant associations between dietary macronutrient intake between 8–12 weeks of gestation and offspring birth weight, but went on to describe that these associations had weakened and/or disappeared when considering maternal dietary intake in the 2nd trimester, assessed between gestational weeks 13–27 [26]. Other studies have published maternal dietary data only from one time-point during pregnancy [12,29] or have calculated average dietary intake values from food frequency questionnaires during pregnancy [9]. The aforementioned studies highlight the need for further research that explores which time window the fetus may be most sensitive to maternal nutrition. Such information may be valuable to give temporally adapted recommendations for pregnant women in the future.

In addition to the analysis of maternal dietary intake both in early and late gestation, our study has several strengths. We utilized multiple measurement approaches, with both direct and indirect measurement tools, to more accurately assess body composition. While most studies considered the effects in the short-term, mostly at birth, our study pursued a follow-up program from birth onwards, with three subsequent time points of examination, to repeatedly monitor child clinical outcomes (i.e., 1, 3, and 5 years of life) within the same group of mother-child pairs. However, there are also some limitations which are worth mentioning. Our sample size was relatively small with considerable attrition from the original cohort. Moreover, only a small subset of 5-year-old children underwent MRI. These factors may suggest selection bias in our cohort and our results should be interpreted with caution. In addition, although we collected information about the general health of the children during the follow-up period via questionnaires, we did not obtain specific information about chronic illnesses or diseases which could influence their growth and development in early childhood. Further, women in our study were relatively well educated and had a health-conscious lifestyle. The percentage of overweight and obese women in our sample was 17.7%, which is lower than in the general population, with prevalence rates of overweight and obesity at 30% (18–29 years of age) and 38% (30–39 years of age), respectively [37]. Therefore, our results are not generalizable. However, we have adjusted for pre-pregnancy BMI in our models. Furthermore, we have determined macronutrient intake with estimated dietary records, a method that has been criticized because it relies on self-reported data and the potential for inaccuracy in terms of under- and over-reporting is considerable [38]. However, we made a particular effort to control reporting bias by providing personal dietary counselling beforehand to accurately estimate maternal dietary intake, which may allow a more precise assessment compared to the commonly used food frequency questionnaires. It is worth noting that for the associations between maternal diet and abdominal subcutaneous and preperitoneal fat at birth, we used ultrasound data that have been assessed at 6 weeks postpartum (due to feasibility reasons), while all other anthropometric data were assessed immediately after birth (weight, height) or 3–5 days postpartum (SFT measurements). This may have altered US-outcome of Table 3 and Table 5 and respective Tables in the Online Supplemental Material. Finally, although we adjusted for several maternal and child confounders in our models, we cannot exclude the possibility that residual confounding may have affected our results. 

## 5. Conclusions

Maternal diet during pregnancy is assumed to be an influencing and possibly modifiable risk factor for future offspring health outcomes. Our study participants kept on average a rather healthy diet in accordance with current recommendations for a balanced dietary intake during pregnancy [39]. Our findings are to some extent questioning the concept of a low-fat, high-carbohydrate and fiber-rich diet, particularly during late pregnancy. Albeit statistical significance was not often attained, our results may suggest that a higher fat intake, in particular PUFAs, as well as lower sugar and carbohydrate consumption may have a potential protective effect regarding the risk of early childhood obesity. Our data also highlight the need for systematic dietary intervention studies in pregnant women to investigate the effect of maternal macronutrient composition on offspring body composition and fat distribution in the short- and the long-term. A better understanding of the link between dietary intake during pregnancy and offspring body composition may promise new opportunities for a primary prevention strategy against childhood obesity.

## Figures and Tables

**Table 1:** Characteristics of mother-child pairs at early and late gestation ^1^.

Maternal Characteristics		Early Gestation	Late Gestation
Age, year		31.92 ± 4.64 (186) ^2^	31.90 ± 4.60 (167)
BMI before pregnancy, kg/m^2^		22.23 ± 2.92 (186)	22.26 ± 2.96 (167)
BMI classification ^3^			
Underweight, *n* (%)		10 (5.4)	9 (5.4)
Normal weight, *n* (%)		143 (76.9)	128 (76.6)
Overweight, *n* (%)		30 (16.1)	27 (16.2)
Obese, *n* (%)		3 (1.6)	3 (1.8)
Gestational age, week		39.64 ± 1.48 (186)	39.74 ± 1.30 (167)
Primiparae, *n* (%)		110 (59.1)	98 (58.7)
Offspring characteristics			
At birth	Male, *n* (%)	97 (52.2)	85 (50.9)
	Weight, kg	3.44 ± 0.52 (186)	3.47 ± 0.48 (167)
	zBMI ^4^	0.18 ±1.04 (186)	0.21 ± 0.97 (167)
	Fat mass ^5^, kg	0.49 ± 0.14 (166)	0.49 ± 0.14 (151)
	Subcutaneous fat area_axial_^6^, mm^2^	30.8 ± 12.2 (159)	30.62 ± 12.28 (148)
	Preperitoneal fat area_axial_^6^, mm^2^	10.7 ± 3.5 (151)	10.78 ± 3.56 (141)
At 1 year	Male, *n* (%)	85 (50.6)	80 (50.6)
	Weight, kg	9.53 ± 1.04 (168)	9.53 ± 1.05 (158)
	zBMI	0.09 ± 1.01 (168)	0.11 ± 1.00 (158)
	Fat mass, kg	1.90 ± 0.43 (163)	1.89 ± 0.43 (153)
	Subcutaneous fat area_axial_, mm^2^	28.39 ± 13.43 (154)	28.15 ± 13.40 (146)
	Preperitoneal fat area_axial_, mm^2^	17.77 ± 5.89 (153)	17.82 ± 5.95 (145)
At 3 years	Male, *n* (%)	82 (51.3)	77 (51.3)
	Weight, kg	14.60 ± 1.68 (160)	14.57 ± 1.74 (150)
	zBMI	0.15 ± 2.14 (160)	0.18 ± 2.21 (150)
	Fat mass, kg	2.71 ± 0.60 (112)	2.72 ± 0.61 (103)
	Subcutaneous fat area_axial_, mm^2^	19.75 ± 12.02 (101)	19.68 ±12.12 (93)
	Preperitoneal fat area_axial_, mm^2^	32.63 ± 11.30 (100)	32.55 ± 11.44 (92)
At 5 years	Male, *n* (%)	77 (50.7)	73 (50.7)
	Weight, kg	18.99 ± 2.48 (152)	18.96 ± 2.54 (144)
	zBMI	−0.13 ± 0.82 (152)	−0.14 ± 0.83 (144)
	Fat mass, kg	3.47 ± 1.00 (111)	3.47 ± 1.02 (105)
	Subcutaneous fat area_axial_, mm^2^	20.87 ± 12.84 (96)	20.63 ± 12.77 (91)
	Preperitoneal fat area_axial_, mm^2^	48.52 ± 14.19 (95)	48.29 ± 14.56 (90)
	SAT volume, cm^3^	563.51 ± 155.35 (44)	565.82 ± 158.64 (42)
	VAT volume, cm^3^	104.19 ± 33.71 (44)	104.81 ± 34.40 (42)

^1^ Early gestation ≙ 15th week of gestation, late gestation ≙ 32nd week of gestation; SAT, subcutaneous adipose tissue; VAT, visceral adipose tissue; zBMI, BMI *z* score. ^2^ Mean ± SD; *n* in parentheses (all such values). Values were calculated from the observed data. ^3^ BMI classification according to World Health Organization [24]. ^4^ zBMI according to [19]. ^5^ Calculated out of the sum of 4 SFTs (biceps + triceps + subscuapular + suprailiac) according to Weststrate and Deurenberg [20]. ^6^ Ultrasound measurements were performed at 6 weeks postpartum.

**Table 2:** Maternal dietary intake at early and late gestation ^1^.

	Early Gestation ^2^ (*n* = 186)	Late Gestation ^2^ (*n* = 167)	*p* ^3^
Energy, kcal/day	2156.9 ± 375.3	2207.6 ± 460.3	0.384
Energy, kJ/day	9030.4 ± 1571.4	9242.8 ± 1927.2	0.384
Fat, g/day	79.8 ± 21.1	83.5 ± 23.5	0.137
SFA, g/day	37.5 ± 10.2	36.6 ± 12.7	0.069
MUFA, g/day	26.1 ± 7.7	27.2 ± 8.4	0.168
PUFA, g/day	13.2 ± 5.5	13.6 ± 5.1	0.769
Carbohydrate, g/day	270.8 ± 56.0	276.0 ± 67.8	0.759
Sugar, g/day	131.3 ± 42.3	140.1 ± 50.2	0.068
Fiber, g/day	24.5 ± 6.7	25.1 ± 7.9	0.493
Protein, g/day	80.1 ± 17.4	79.1 ± 18.1	0.669
Fat, % of energy	34.3 ± 6.2	35.1 ± 6.0	0.087
SFA, % of energy	14.8 ± 3.3	15.3 ± 3.7	0.050
MUFA, % of energy	10.9 ± 2.4	11.1± 2.2	0.188
PUFA, % of energy	5.7 ± 2.1	5.8 ± 2.1	0.794
Carbohydrate, % of energy	51.5 ± 6.2	51.2 ± 6.4	0.305
Sugar, % of energy	24.4 ± 6.3	25.1 ± 6.5	0.229
Fiber, % of energy	2.3 ± 0.6	2.3 ± 0.6	0.939
Protein, % of energy	15.3 ± 2.7	14.8 ± 2.6	0.132

^1^ Analysis of 3 out of 7-day dietary records, randomly chosen by a customized Microsoft Access software, while the first day was rejected (1 weekend day, 2 weekdays). ^2^ Early gestation ≙ 15th week of gestation, late gestation ≙ 32nd week of gestation. ^3^ Comparisons between time points were tested with a paired t-test.

**Table 3:** Partition model from early gestation (15th week of gestation) at birth, 1 year, 3 years, and 5 years ^1^.

Body Composition Parameter	Macronutrients	Birth ^2^			1 Year			3 Years			5 Years		
		*n*	Beta (95% CI)	*p*	*n*	Beta (95% CI)	*p*	*n*	Beta (95% CI)	*p*	*n*	Beta (95% CI)	*p*
Weight, g	Fat	186	22.99 (−11.32, 57.30)	0.188	168	38.44 (−43.57, 120.46)	0.356	160	6.98 (−132.65, 146.61)	0.921	152	1.14 (−208.97, 211.25)	0.991
	SFA	186	42.03 (−37.11, 121.18)	0.296	168	67.76 (−122.18, 257.69)	0.482	160	17.50 (−307.37, 342.36)	0.915	152	159.25 (−334.70, 653.19)	0.525
	MUFA	186	67.88 (−44.52, 180.28)	0.235	168	161.82 (−111.41, 435.05)	0.244	160	82.73 (−390.44, 555.89)	0.730	152	100.36 (−603.06, 815.77)	0.767
	PUFA	186	5.42 (−125.42, 136.26)	0.935	168	−2.54 (−312.83, 307.74)	0.987	160	−94.56 (−639.56, 450.43)	0.732	152	−509.17 (−1336.75, 318.42)	0.226
	Carbohydrate	186	−1.17 (−30.95, 28.61)	0.939	168	−1.21 (−72.87, 70.46)	0.974	160	5.52 (−119.10, 130.14)	0.930	152	−5.67 (−197.95, 186.61)	0.954
	Sugar	185	−12.86 (−51.36, 25.64)	0.511	167	−16.76 (−107.79, 74.27)	0.717	159	−4.72 (−161.87, 152.44)	0.953	151	−16.89 (−260.74, 226.96)	0.891
	Fiber	186	43.38 (−64.72, 151.48)	0.429	168	80.71 (−184.00, 345.42)	0.548	160	149.35 (−309.40, 609.30)	0.520	152	−4.35 (−729.09, 720.40)	0.991
	Protein	186	3.76 (−101.13, 108.65)	0.944	168	−60.18 (−316.58, 196.22)	0.644	160	50.40 (−394.05, 494.85)	0.823	152	102.05 (−575.07, 779.17)	0.766
zBMI ^3^	Fat	186	0.07 (−0.01, 0.14)	0.072	168	0.04 (−0.05, 0.12)	0.388	160	0.14 (−0.04, 0.31)	0.127	152	0.00 (−0.07, 0.07)	0.944
	SFA	186	0.09 (−0.08, 0.26)	0.306	168	0.05 (−0.15, 0.24)	0.650	160	0.282 (−0.13, 0.69)	0.173	152	0.09 (−0.07, 0.25)	0.248
	MUFA	186	0.28 (0.04, 0.52)	0.021	168	0.17 (−0.10, 0.45)	0.219	160	0.28 (−0.31,0.87)	0.346	152	0.06 (−0.17, 0.29)	0.610
	PUFA	186	0.04 (−0.24, 0.32)	0.764	168	−0.02 (−0.33, 0.30)	0.919	160	0.43 (−0.25, 1.11)	0.209	152	−0.21 (−0.48, 0.06)	0.126
	Carbohydrate	186	−0.01 (−0.07, 0.06)	0.805	168	0.02 (−0.05, 0.10)	0.552	160	−0.07 (−0.22, 0.09)	0.412	152	−0.01 (−0.06, 0.07)	0.824
	Sugar	185	−0.04 (−0.12, 0.04)	0.326	167	−0.01 (−0.10, 0.09)	0.896	159	−0.04 (−0.24, 0.16)	0.683	151	0.00 (−0.08, 0.08)	0.929
	Fiber	186	0.13 (−0.10, 0.36)	0.264	168	0.12 (−0.15, 0.39)	0.388	160	−0.18 (−0.76, 0.40)	0.548	152	−0.04 (−0.27, 0.20)	0.764
	Protein	186	−0.03 (−0.25, 0.20)	0.823	168	−0.15 (−0.41, 0.12)	0.272	160	−0.409 (−0.97, 0.15)	0.148	152	−0.15 (−0.36, 0.07)	0.192
Fat mass, g	Fat	166	4.67 (−6.70, 16.31)	0.429	163	16.10 (−20.02, 52.23)	0.380	112	14.68 (−48.87, 78.22)	0.648	111	43.27 (−59.20, 145.73)	0.404
	SFA	166	14.31 (−12.98, 41.59)	0.302	163	25.58 (−57.86, 109.03)	0.546	112	70.21 (−72.67, 213.08)	0.332	111	161.24 (−69.77, 392.25)	0.169
	MUFA	166	8.71 (−29.37, 46.79)	0.652	163	76.0 (−43.50, 195.49)	0.211	112	33.94 (−171.31, 239.20)	0.744	111	100.36 (−231.64, 432.35)	0.550
	PUFA	166	−7.85 (−51.60, 35.91)	0.724	163	−46.04 (−180.68, 88.60)	0.500	112	−67.18 (−317.85, 183.48)	0.596	111	4.59 (−406.39, 415.56)	0.982
	Carbohydrate	166	−0.57 (−10.69, 9.55)	0.912	163	14.57 (−16.80, 45.93)	0.360	112	5.05 (−49.59, 59.68)	0.855	111	−44.42 (−133.60, 44.75)	0.326
	Sugar	166	−5.55 (−18.72, 7.64)	0.406	162	16.42 (−23.17, 56.01)	0.414	111	1.10 (−64.65, 66.84)	0.974	110	−52.22 (−159.41, 54.96)	0.336
	Fiber	166	20.66 (−17.66, 58.99)	0.289	163	32.22 (−86.09, 150.53)	0.591	112	−26.54 (−238.87, 185.78)	0.805	111	−148.73 (−496.77, 199.30)	0.399
	Protein	166	3.40 (−31.95, 38.75)	0.850	163	−22.62 (−134.67, 89.44)	0.691	112	−110.23 (−314.81, 94.36)	0.288	111	−5.92 (−340.12, 328.27)	0.972
Subcutaneous fat area_sagittal_^4^, mm^2^	Fat	159	−0.17 (−1.20, 0.87)	0.749	154	−1.04 (−2.19, 0.12)	0.078	101	−0.59 (−1.85, 0.67)	0.353	96	−0.94 (−2.32, 0.43)	0.177
	SFA	159	−0.04 (−2.43, 2.36)	0.976	154	−2.08 (−4.75, 0.59)	0.126	101	0.10 (−2.84, 3.04)	0.947	96	−0.31 (−3.47, 2.86)	0.849
	MUFA	159	0.54 (−2.81, 3.89)	0.751	154	−1.13 (−4.99, 2.74)	0.564	101	−1.60 (−5.81, 2.61)	0.453	96	−1.18 (−5.80, 3.43)	0.612
	PUFA	159	−3.04 (−6.88, 0.80)	0.120	154	−3.64 (−7.92, 0.64)	0.095	101	−2.19 (−7.11, 2.72)	0.378	96	−3.83 (−9.25, 1.60)	0.164
	Carbohydrate	159	0.33 (−0.55, 1.22)	0.456	154	0.76 (−0.25, 1.76)	0.141	101	0.01 (−1.14, 1.16)	0.988	96	−0.36 (−1.58, 0.86)	0.561
	Sugar	159	0.86 (−0.26, 1.99)	0.131	153	0.72 (−0.52, 1.96)	0.255	100	0.45 (−0.82, 1.71)	0.488	95	−0.07 (−1.31, 1.44)	0.924
	Fiber	159	0.96 (−2.41, 4.32)	0.575	154	0.27 (−3.51, 4.05)	0.889	101	−1.44 (−5.73, 2.84)	0.506	96	−3.62 (−8.29, 1.05)	0.127
	Protein	159	0.62 (−2.46, 3.70)	0.692	154	−2.55 (−6.14, 1.03)	0.162	101	−2.70 (−6.66, 1.27)	0.180	96	−2.57 (−7.13, 1.99)	0.266
Preperitoneal fat area_sagittal_^4^, mm^2^	Fat	151	−0.11 (−0.43, 0.20)	0.471	153	−0.30 (−0.82, 0.21)	0.247	100	−0.35 (−1.61, 0.91)	0.581	95	−1.54 (−3.09, 0.02)	0.052
	SFA	151	−0.12 (−0.84, 0.60)	0.741	153	−0.60 (−1.79, 0.59)	0.321	100	−0.08 (−3.02, 2.87)	0.958	95	−2.16 (−5.79, 1.48)	0.241
	MUFA	151	0.20 (−0.82, 1.22)	0.700	153	−0.07 (−1.78, 1.65)	0.938	100	−0.90 (−5.10, 3.31)	0.673	95	−4.35 (−9.65, 0.95)	0.106
	PUFA	151	−0.92 (−2.09, 0.25)	0.123	153	−0.92 (−2.83, 0.99)	0.341	100	−2.21 (−7.10, 2.69)	0.373	95	−5.36 (−11.53, 0.81)	0.088
	Carbohydrate	151	0.01 (−0.26, 0.28)	0.966	153	−0.05 (−0.51, 0.40)	0.815	100	0.28 (−0.87, 1.43)	0.632	95	0.90 (−0.51, 2.31)	0.208
	Sugar	151	−0.11 (−0.45, 0.23)	0.530	152	−0.34 (−0.89, 0.22)	0.235	99	−0.27 (−1.58, 1.04)	0.684	94	−0.60 (−2.23, 1.03)	0.465
	Fiber	151	0.13 (−0.89, 1.14)	0.803	153	−0.35 (−2.03, 1.34)	0.686	100	−0.95 (−5.22, 3.32)	0.661	95	1.64 (−3.76, 7.04)	0.547
	Protein	151	0.24 (−0.72, 1.19)	0.626	153	0.07 (−1.53, 1.67)	0.931	100	−0.81 (−4.78, 3.17)	0.688	95	−1.08 (−6.38, 4.21)	0.685

^1^ Beta (95% confidence interval (CI)) illustrates the estimated unit change in the respective body composition parameter, associated with a 100 kcal increase of the macronutrient of interest (non-isocaloric); exception: for fiber, beta (95% CI) illustrates the estimated unit change in the respective body composition parameter, associated with a 20 kcal increase (≙10 g) of fiber (non-isocaloric); regressions were adjusted for sex (except zBMI), pre-pregnancy BMI, gestational age in days, and group allocation; zBMI, BMI *z* score. ^2^ Ultrasound measurements were performed at 6 week postpartum. ^3^ zBMI according to [19]. ^4^ Subcutaneous and preperitoneal fat were measured as areas of 1-cm length in sagittal plane in the middle of the xiphoid process according to Brei et al. [21].

**Table 4:** Partition model from early (15th week of gestation) and late gestation (32nd week of gestation) on offspring abdominal subcutaneous and visceral adipose tissue volume at 5 years ^1^.

Body Composition Parameter	Macronutrients	Early Gestation			Late Gestation		
		*n*	Beta (95% CI)	*p*	*n*	Beta (95% CI)	*p*
SAT volume, cm^3^	Fat	44	11.89 (−12.13, 35.90)	0.323	42	−15.47 (−44.95, 14.02)	0.294
	SFA	44	37.27 (−13.79, 88.34)	0.148	42	−16.89 (−80.54, 46.77)	0.594
	MUFA	44	11.55 (−75.25, 98.34)	0.789	42	13.27 (−87.95, 114.49)	0.792
	PUFA	44	3.33 (−93.09, 99.75)	0.945	42	−104.74 (−216.54, 7.05)	0.065
	Carbohydrate	44	−4.36 (−29.32, 20.59)	0.725	42	4.38 (−18.61, 27.36)	0.701
	Sugar	43	−10.23 (−40.84, 20.39)	0.502	42	21.17 (−9.83, 52.16)	0.174
	Fiber	44	−53.30 (−147.22, 40.63)	0.258	42	28.40 (−39.38, 96.18)	0.401
	Protein	44	−54.73 (−138.33, 28.86)	0.193	42	−6.93 (−111.90, 98.04)	0.894
VAT volume, cm^3^	Fat	44	−2.03 (−7.39, 3.34)	0.449	42	−1.56 (−8.17, 5.05)	0.634
	SFA	44	0.51 (−11.05, 12.08)	0.929	42	−0.38 (−14.53, 13.77)	0.956
	MUFA	44	−9.72 (−28.65, 9.21)	0.305	42	5.82 (−16.54, 28.17)	0.601
	PUFA	44	−8.91 (−30.00, 12.18)	0.398	42	−12.29 (37.98, 13.40)	0.338
	Carbohydrate	44	0.35 (−5.13, 5.84)	0.897	42	−1.22 (−6.38, 3.94)	0.634
	Sugar	43	0.13 (−6.86, 7.12)	0.970	42	0.97 (−6.17, 8.10)	0.785
	Fiber	44	−20.71 (−40.64, −0.78)	0.042	42	1.23 (−13.96, 16.42)	0.870
	Protein	44	−11.42 (−29.99, 7.15)	0.221	42	−0.15 (−23.43, 23.14)	0.990

^1^ Beta (95% confidence interval (CI)) illustrates the estimated unit change in the respective body composition parameter, associated with a 100 kcal increase of the macronutrient of interest (non-isocaloric); exception: for fiber, beta (95% CI) illustrates the estimated unit change in the respective body composition parameter, associated with a 20 kcal increase (≙10 g) of fiber (non-isocaloric); regressions were adjusted for sex (except zBMI), pre-pregnancy BMI, gestational age in days, and group allocation; SAT, subcutaneous adipose tissue; VAT, visceral adipose tissue; zBMI, BMI *z* score.

**Table 5:** Partition model from late gestation (32nd week of gestation) at birth, 1 year, 3 years, and 5 years ^1^.

Body Composition Parameter	Macronutrients	Birth ^2^			1 Year			3 Years			5 Years		
		*n*	Beta (95% CI)	*p*	*n*	Beta (95% CI)	*p*	*n*	Beta (95% CI)	*p*	*n*	Beta (95% CI)	*p*
Weight, g	Fat	167	−20.87 (−55.62, 13.89)	0.237	158	−10.64 (−93.47, 72.18)	0.800	150	10.78 (−133.40, 154.95)	0.883	144	−6.51 (−218.95, 205.94)	0.952
	SFA	167	0.11 (−69.05, 69.27)	0.998	158	−69.37 (−232.83, 94.10)	0.403	150	−47.63 (−334.87, 239.60)	0.744	144	−61.44 (−488.00, 365.08)	0.776
	MUFA	167	−37.07 (−158.02, 83.89)	0.546	158	154.51 (−132.57, 441.59)	0.288	150	250.85 (−250.06, 751.76)	0.324	144	335.58 (−406.27, 1077.44)	0.373
	PUFA	167	−161.35 (−312.25, −10.44)	0.036	158	87.62 (−276.82, 452.05)	0.635	150	227.09 (−400.75, 854.93)	0.476	144	143.39 (−806.10, 1092.89)	0.766
	Carbohydrate	167	7.52 (−19.19, 34.23)	0.579	158	−11.75 (−75.50, 51.99)	0.716	150	−44.96 (−158.14, 68.22)	0.434	144	−60.86 (−231.22, 109.50)	0.481
	Sugar	167	28.01 (−7.78, 63.80)	0.124	158	−5.22 (−91.81, 81.37)	0.905	150	−12.71 (−165.38, 139.96)	0.870	144	−50.37(−275.73, 175.00)	0.659
	Fiber	167	38.69 (−56.98, 134.36)	0.426	158	112.83 (−117.02, 342.68)	0.334	150	276.50 (−122.16, 675.17)	0.173	144	416.43 (−203.79, 1036.65)	0.186
	Protein	167	−103.33 (−213.88, 7.22)	0.067	158	−109.29 (−374.43, 155.84)	0.417	150	−188.08 (−652.30, 276.14)	0.425	144	−327.72 (−1032.07, 376.63)	0.359
zBMI ^3^	Fat	167	−0.02 (−0.09, 0.06)	0.674	158	−0.06 (−0.15, 0.02)	0.124	150	−0.15 (0.33, 0.03)	0.109	144	−0.02 (−0.09, 0.05)	0.517
	SFA	167	0.04 (−0.10, 0.19)	0.566	158	−0.16 (−0.32, −0.00)	0.049	150	−0.24 (−0.59, 0.12)	0.187	144	−0.06 (−0.20, 0.07)	0.369
	MUFA	167	−0.02 (−0.28, 0.23)	0.870	158	−0.07 (−0.35, 0.22)	0.640	150	−0.25 (−0.88, 0.37)	0.426	144	0.05 (−0.19, 0.29)	0.709
	PUFA	167	−0.20 (−0.52, 0.12)	0.209	158	0.02 (−0.34, 0.38)	0.921	150	−0.31 (−1.09, 0.47)	0.431	144	−0.00 (−0.30, 0.31)	0.986
	Carbohydrate	167	−0.01 (−0.07, 0.05)	0.707	158	0.03 (−0.04, 0.09)	0.384	150	0.05 (−0.09, 0.19)	0.505	144	0.01 (−0.05, 0.06)	0.774
	Sugar	167	0.02 (−0.06, 0.09)	0.684	158	0.03 (−0.05, 0.12)	0.450	150	0.11 (−0.08, 0.29)	0.269	144	−0.01 (−0.08, 0.07)	0.866
	Fiber	167	0.03 (−0.17, 0.24)	0.751	158	0.14 (−0.08, 0.37)	0.212	150	0.22 (−0.28, 0.72)	0.386	144	0.23 (0.03, 0.45)	0.023
	Protein	167	−0.22 (−0.45, 0.02)	0.070	158	−0.22 (−0.48, 0.04)	0.100	150	−0.65 (−1.22, −0.08)	0.026	144	−0.24 (−0.47, −0.02)	0.033
Fat mass, g	Fat	151	−8.74 (−20.52, 3.04)	0.145	153	−10.90 (−46.68, 24.88)	0.548	103	−20.57 (−85.37, 44.22)	0.530	105	−78.02 (−181.54, 25.49)	0.138
	SFA	151	−1.11 (−24.51, 22.29)	0.926	153	−36.03 (−105.77, 33.71)	0.309	103	−60.68 (−185.97, 64.61)	0.339	105	−137.30 (−337.64, 63.05)	0.177
	MUFA	151	−21.70 (−62.95, 19.56)	0.300	153	39.95 (−84.83, 164.74)	0.526	103	38.78 (−185.85, 263.42)	0.733	105	−9.40 (−364.53, 345.73)	0.958
	PUFA	151	−58.39 (−110.55, −6.24)	0.028	153	−27.84 (−185.07, 129.39)	0.727	103	33.91 (−225.89, 293.71)	0.796	105	−51.04 (−451.71, 349.63)	0.801
	Carbohydrate	151	0.61 (−8.47, 9.69)	0.895	153	5.30 (−21.87, 32.46)	0.701	103	−3.06 (−50.32, 44.20)	0.898	105	−31.13 (−106.00, 43.74)	0.411
	Sugar	151	3.51(−9.08, 16.10)	0.582	153	8.71 (−28.34, 45.75)	0.643	103	0.11 (−59.70, 59.91)	0.997	105	−25.89 (−120.61, 68.82)	0.589
	Fiber	151	14.41 (−19.45, 48.27)	0.402	153	76.99 (−23.50, 177.48)	0.132	103	172.41 (6.43, 338.38)	0.042	105	229.24 (−36.1, 494.57)	0.090
	Protein	151	−10.70 (−48.10, 26.70)	0.573	153	−16.64 (−131.39, 98.10)	0.775	103	−94.94 (−309.97, 120.10)	0.383	105	−230.38 (−571.40, 110.64)	0.183
Subcutaneous fat area_sagittal_^4^, mm^2^	Fat	148	−0.73 (−1.77, 0.31)	0.167	146	−1.30 (−2.40, −0.20)	0.021	93	−1.18 (−2.50, 0.14)	0.79	91	−1.68 (−3.13, −0.23)	0.024
	SFA	148	−1.74 (−3.81, 0.33)	0.098	146	−2.79 (−4.98, −0.60)	0.013	93	−1.80 (−4.43, 0.84)	0.179	91	−1.67 (−4.54, 1.20)	0.249
	MUFA	148	−1.36 (−4.93, 2.20)	0.451	146	−2.18 (−6.10, 1.74)	0.274	93	−0.35 (−4.88, 4.19)	0.880	91	−1.90 (−6.75, 2.95)	0.438
	PUFA	148	−1.53 (−5.99, 2.94)	0.501	146	−1.41 (−6.42, 3.61)	0.580	93	−4.08 (−9.22, 1.06)	0.118	91	−6.16 (−11.70, −0.63)	0.030
	Carbohydrate	148	0.73 (−0.07, 1.52)	0.073	146	0.73 (−0.13, 1.59)	0.095	93	0.23 (−0.74, 1.19)	0.645	91	0.09 (−0.93, 1.11)	0.858
	Sugar	148	0.64 (−0.45, 1.72)	0.248	146	0.47 (−0.70, 1.65)	0.427	93	0.71 (−0.48, 1.90)	0.237	91	0.54 (−0.73, 1.80)	0.400
	Fiber	148	1.49 (−1.39, 4.37)	0.307	146	3.50 (0.36, 6.65)	0.029	93	2.43 (−1.01, 5.87)	0.164	91	1.13 (−2.58, 4.85)	0.546
	Protein	148	0.22 (−3.06, 3.50)	0.893	146	−1.62 (−5.48, 2.24)	0.409	93	−1.20 (−5.15, 2.75)	0.547	91	−3.77 (−8.37, 0.83)	0.107
Preperitoneal fat area_sagittal_^4^, mm^2^	Fat	141	−0.05 (−0.38, 0.27)	0.746	145	0.03 (−0.48, 0.54)	0.918	92	−0.22 (−1.55, 1.11)	0.747	90	−1.48 (−3.17, 0.21)	0.085
	SFA	141	−0.04 (−0.69, 0.61)	0.903	145	−0.58 (−1.58, 0.43)	0.261	92	−0.02 (−2.66, 2.63)	0.991	90	−2.14 (−5.46, 1.17)	0.201
	MUFA	141	−0.05 (−1.14, 1.05)	0.936	145	0.90 (−0.88, 2.67)	0.321	92	−0.18 (−4.71, 4.34)	0.936	90	−3.56 (−9.18, 2.07)	0.212
	PUFA	141	−0.40 (−1.79, 0.99)	0.572	145	1.55 (−0.71, 3.80)	0.177	92	−1.24 (−6.39, 3.92)	0.634	90	−1.69 (−8.24, 4.87)	0.610
	Carbohydrate	141	−0.05 (−0.30, 0.20)	0.694	145	−0.11 (−0.51, 0.28)	0.576	92	−0.16 (−1.15, 0.82)	0.745	90	−0.03 (−1.22, 1.17)	0.967
	Sugar	141	−0.06 (−0.40, 0.28)	0.733	145	0.02 (−0.51, 0.56)	0.932	92	0.41 (−0.79, 1.62)	0.497	90	0.77 (−0.70, 2.23)	0.301
	Fiber	141	0.15 (−0.74, 1.04)	0.738	145	0.04 (−1.40, 1.49)	0.952	92	−0.32 (−3.80, 3.17)	0.857	90	0.48 (−3.83, 4.79)	0.825
	Protein	141	0.18 (−0.81, 1.18)	0.715	145	1.06 (−0.68, 2.79)	0.231	92	−0.89 (−4.84, 3.06)	0.654	90	−2.21 (−7.68, 3.25)	0.423

^1^ Beta (95% confidence interval (CI)) illustrates the estimated unit change in the respective body composition parameter, associated with a 100 kcal increase of the macronutrient of interest (non-isocaloric); exception: for fiber, beta (95% CI) illustrates the estimated unit change in the respective body composition parameter, associated with a 20 kcal increase (≙10 g) of fiber (non-isocaloric); regressions were adjusted for sex (except zBMI), pre-pregnancy BMI, gestational age in days, and group allocation; zBMI, BMI *z* score. ^2^ Ultrasound measurements were performed at 6 week postpartum. ^3^ zBMI according to [19]. ^4^ Subcutaneous and preperitoneal fat were measured as areas of 1-cm length in sagittal plane in the middle of the xiphoid process according to Brei et al. [21].

## Supplementary Materials

The following are available online at http://www.mdpi.com/2072-6643/10/5/579/s1, Figure S1: Flowchart of mother-child pairs with available data at each time point. Table S1: Substitution model from early gestation (15th week of gestation) at birth, 1 year, 3 years, and 5 years. Table S2: Substitution model from early (15th week of gestation) and late gestation (32nd week of gestation) on offspring abdominal subcutaneous and visceral adipose tissue volume at 5 years. Table S3: Substitution model from late gestation (32nd week of gestation) at birth, 1 year, 3 years, and 5 years.
