# Supplementary material for: Impact of Dietary Macronutrient Intake during Early and Late Gestation on Offspring Body Composition at Birth, 1, 3, and 5 Years of Age
Source: Nutrients. 2018 May 8;10(5):579. doi: 10.3390/nu10050579 (PMC5986459; doi:10.3390/nu10050579)
Supplement: Supplementary file 1 [file nutrients-10-00579-s001.pdf]

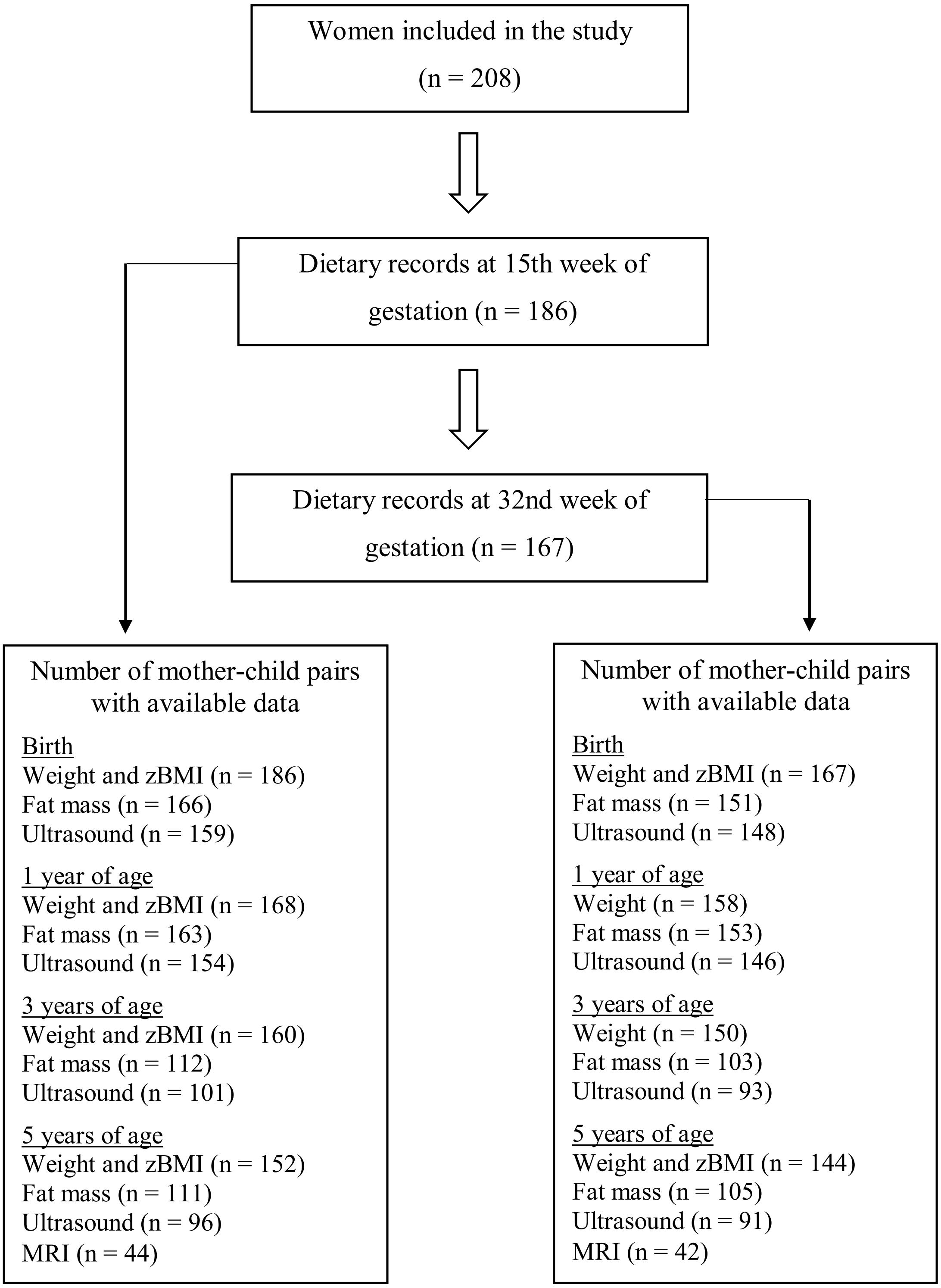

SUPPLEMENTAL FIGURE 1 Flowchart of mother-child pairs with available data at each time point. Data for fat mass, ultrasound, and MRI were not available for all children as indicated in parentheses. For the associations between maternal diet and abdominal subcutaneous and preperitoneal fat at birth, we used ultrasound data that have been performed at 6 weeks postpartum. zBMI, BMI *z* score.

ONLNE SUPPLEMENTAL MATERIAL

SUPPLEMENTAL TABLE 1 Substitution model from early gestation (15th week of gestation) at birth, 1 year, 3 years, and 5 years[1]

Body Composition Parameter	Macronutrients	Birth [2]			1 year			3 years			5 years		
		n	Beta (95% CI)	*p*	n	Beta (95% CI)	*p*	n	Beta (95% CI)	*p*	n	Beta (95% CI)	*p*
Weight, g	Fat	186	3.08 (−7.37, 13.53)	0.562	168	6.80 (−18.60, 32.20)	0.598	160	−5.19 (−49.21, 38.83)	0.816	152	−3.89 (−70.10, 62.32)	0.908
	SFA	186	3.23 (−16.68, 23.14)	0.749	168	12.43 (−35.53, 60.39)	0.609	160	0.91 (−81.55, 83.37)	0.983	152	37.80 (−87.97, 163.56)	0.553
	MUFA	186	10.91 (−16.14, 37.97	0.427	168	21.12 (−45.17, 87.40)	0.530	160	−9.71 (−126.25, 106.83)	0.869	152	−1.94 (−177.78, 173.90)	0.983
	PUFA	186	0.64 (−29.28, 30.56)	0.966	168	0.05 (−71.20, 71.30)	0.999	160	−25.90 (−151.10, 99.31)	0.683	152	−108.39 (−294.64, 77.86)	0.252
	Carbohydrate	186	−2.60 (−13.02, 7.82)	0.623	168	−2.46 (−27.85, 22.92)	0.848	160	4.45 (−39.05, 47.94)	0.840	152	2.13 (−64.03, 68.29)	0.949
	Sugar	185	−6.08 (−16.12, 3.97)	0.234	167	−11.15 (−34.90, 12.60)	0.355	159	−6.34 (−47.13, 34.44)	0.759	151	−10.62 (−73.02, 51.79)	0.737
	Fiber	186	47.44 (−65.20, 160.10)	0.407	168	97.64 (−181.09, 376.38)	0.490	160	169.50 (−314.10, 653.10)	0.490	152	26.79 (−726.10, 779.69)	0.944
	Protein	186	2.49 (−21.76, 26.74)	0.840	168	−16.18 (−74.87, 42.50)	0.587	160	14.09 (−87.14, 115.31)	0.784	152	26.63 (−127.00, 180.26)	0.732
zBMI[3]	Fat	186	0.01 (−0.01, 0.04)	0.241	168	0.01 (−0.02, 0.03)	0.708	160	0.04 (−0.02, 0.09)	0.165	152	0.00 (−0.02, 0.02)	0.993
	SFA	186	0.01 (−0.03, 0.05)	0.631	168	0.00 (−0.05, 0.05)	0.869	160	0.06 (−0.04, 0.17)	0.234	152	0.02 (−0.02, 0.06)	0.306
	MUFA	186	0.06 (0.00, 0.11)	0.053	168	0.03 (−0.04, 0.09)	0.463	160	0.06(−0.09, 0.20)	0.458	152	0.01 (−0.05, 0.06)	0.773
	PUFA	186	0.01 (−0.06, 0.07)	0.837	168	0.00 (−0.07, 0.07)	0.984	160	0.08 (−0.07, 0.24)	0.294	152	−0.04 (−0.10, 0.02)	0.151
	Carbohydrate	186	−0.01 (−0.03, 0.01)	0.414	168	0.00 (−0.02, 0.03)	0.766	160	−0.03 (−0.08, 0.03)	0.345	152	0.01 (−0.02, 0.03)	0.621
	Sugar	185	−0.02 (−0.04, 0.00)	0.113	167	−0.01 (−0.03, 0.02)	0.491	159	−0.01 (−0.06, 0.04)	0.665	151	0.00 (−0.02, 0.02)	0.876
	Fiber	186	0.12 (−0.14, 0.35)	0.387	168	0.13 (−0.15, 0.42)	0.362	160	−0.15 (−0.76, 0.47)	0.638	152	0.01 (−0.23, 0.25)	0.934
	Protein	186	−0.01 (−0.06, 0.05)	0.843	168	−0.04 (−0.10, 0.02)	0.227	160	−0.08 (−0.21, 0.05)	0.205	152	−0.03 (−0.08, 0.02)	0.310
Fat mass, g	Fat	166	0.46 (−3.07, 3.99)	0.798	163	0.13 (−11.04, 11.31)	0.981	112	3.39 (−15.74, 22.52)	0.726	111	16.40 (−14.53, 47.33)	0.295
	SFA	166	1.28 (−5.58, 8.14)	0.713	163	2.68 (−18.37, 23.73)	0.802	112	17.07 (−18.59, 52.73)	0.345	111	40.90 (−16.67, 98.48)	0.162
	MUFA	166	1.31 (−7.85, 10.47)	0.163	163	9.60 (−19.50, 38.70)	0.516	112	3.81 (−45.36, 52.98)	0.878	111	21.17 (−59.08, 101.41)	0.602
	PUFA	166	−2.22 (−12.25, 7.80)	0.662	163	−13.22 (−44.24, 17.79)	0.401	112	−17.08 (−72.50, 38.34)	0.543	111	3.48 (−86.95, 93.90)	0.939
	Carbohydrate	166	−0.38 (−3.91, 3.16)	0.834	163	2.05 (−9.18, 13.28)	0.719	112	2.58 (−16.80, 21.97)	0.792	111	−13.92 (−45.69, 17.85)	0.387
	Sugar	166	−1.82 (−5.27, 1.64)	0.301	162	1.03 (−9.35, 11.40)	0.845	111	0.50 (−16.24, 17.24)	0.953	110	−11.72 (−38.85, 15.41)	0.394
	Fiber	166	21.51 (−18.53, 61.55)	0.290	163	33.91 (−92.46, 160.29)	0.597	112	−33.83 (−248.70, 181.05)	0.756	111	−142.31 (−495.91, 211.29)	0.427
	Protein	166	1.10 (−7.16, 9.37)	0.792	163	−8.68 (−34.42, 17.06)	0.506	112	−25.51 (−70.98, 19.96)	0.268	111	0.52 (−73.68, 74.73)	0.989
Subcutaneous fat area$_{sagittal}$[4], mm^2	Fat	159	−0.11 (−0.42, 0.21)	0.511	154	−0.28 (−0.63, 0.07)	0.116	101	−0.08 (−0.47, 0.30)	0.671	96	−0.09 (−0.51, 0.32)	0.658
	SFA	159	−0.04 (−0.65, 0.57)	0.895	154	−0.46 (−1.14, 0.21)	0.177	101	0.07 (−0.65, 0.80)	0.843	96	0.06 (−0.73, 0.85)	0.883
	MUFA	159	0.08 (−0.74, 0.83)	0.849	154	−0.29 (−1.22, 0.63)	0.534	101	−0.37 (−1.39, 0.65)	0.476	96	−0.18 (−1.27, 0.92)	0.751
	PUFA	159	−0.695 (−1.56, 0.17)	0.144	154	−0.68 (−1.67, 0.30)	0.173	101	−0.38 (−1.50, 0.0.74)	0.501	96	−0.68 (−1.91, 0.55)	0.275
	Carbohydrate	159	0.08 (−0.23, 0.40)	0.598	154	0.40 (0.05, 0.75)	0.025	101	0.22 (−0.18, 0.61)	0.277	96	0.18 (−0.25, 0.60)	0.413
	Sugar	159	0.20 (−0.09, 0.50)	0.174	153	0.26 (0.07, 0.58)	0.116	100	0.29 (−0.03, 0.61)	0.073	95	0.26 (−0.09, 0.60)	0.143
	Fiber	159	1.05 (−2.54, 4.64)	0.564	154	0.28 (−3.72, 4.27)	0.891	101	−1.19 (−5.58, 3.19)	0.590	96	−2.89 (−7.60, 1.83)	0.227
	Protein	159	0.11 (−0.61, 0.82)	0.768	154	−0.59 (−1.43, 0.26)	0.171	101	−0.52 (−1.37, 0.34)	0.232	96	−0.37 (−1.36, 0.62)	0.459
Preperitoneal fat area$_{sagittal}$[4], mm^2	Fat	151	−0.02 (−0.12, 0.08)	0.722	153	−0.06 (−0.21, 0.10)	0.460	100	−0.11 (−0.50, 0.27)	0.559	95	−0.44 (−0.92, 0.03)	0.064
	SFA	151	−0.02 (−0.20, 0.16)	0.831	153	−0.12 (−0.42, 0.18)	0.422	100	−0.06 (−0.78, 0.67)	0.875	95	−0.49 (−1.40, 0.42)	0.284
	MUFA	151	0.05 (−0.20, 0.30)	0.671	113	−0.01 (−0.42, 0.40)	0.958	100	−0.24 (−1.26, 0.78)	0.638	95	−1.04 (−2.30, 0.21)	0.103
	PUFA	151	−0.14 (−0.40, 0.13)	0.305	153	−0.19 (−0.63, 0.25)	0.390	100	−0.46 (−1.58, 0.65)	0.409	95	−1.12 (−2.52, 0.28)	0.115
	Carbohydrate	151	0.00 (−0.10, 0.10)	0.971	153	0.06 (−0.10, 0.22)	0.479	100	0.15 (−0.25, 0.54)	0.462	95	0.48 (−0.01, 0.954)	0.053
	Sugar	151	−0.03 (−0.12, 0.06)	0.559	152	−0.04 (−0.18, 0.11)	0.621	99	−0.04 (−0.37, 0.29)	0.822	94	−0.07 (−0.48, 0.34)	0.731
	Fiber	151	0.20 (−0.89, 1.29)	0.716	153	−0.35 (−2.13, 1.44)	0.702	100	−0.81 (−5.18, 3.56)	0.714	95	1.67 (−3.77, 7.10)	0.543
	Protein	151	0.11 (−0.11, 0.33)	0.333	153	0.06 (−0.32, 0.43)	0.760	100	−0.05 (−0.91, 0.81)	0.910	95	−0.02 (−1.16, 1.13)	0.979

[1] Beta (95% confidence interval (CI)) illustrates the estimated unit change in the respective body composition parameter, associated with an increase of 1% of energy of the macronutrient of interest in the substitution of other macronutrients (isocaloric); regressions were adjusted for sex (except zBMI), pre-pregnancy BMI, gestational age in days, and group allocation; zBMI, BMI *z* score.[2] Ultrasound measurements were performed at 6 wk postpartum.[3] zBMI according to (1).[4] Subcutaneous and preperitoneal fat were measured as areas of 1-cm length in sagittal plane in the middle of the xiphoid process according to Brei et al. (2).

SUPPLEMENTAL TABLE 2 Substitution model from early (15th week of gestation) and late gestation (32nd week of gestation) on offspring abdominal subcutaneous and visceral adipose tissue volume at 5 years[1]

Body Composition Parameter	Macronutrients	Early gestation			Late gestation		
		n	Beta (95% CI)	*p*	n	Beta (95% CI)	*p*
SAT volume, cm^3	Fat	44	3.35 (−3.80, 10.49)	0.348	42	−3.45 (12.95, 6.05)	0.465
	SFA	44	8.20 (−4.40, 20.79)	0.195	42	−2.86 (−18.70, 12.97)	0.716
	MUFA	44	1.07 (−19.14, 21.29)	0.915	42	6.37 (−20.14, 32.88)	0.629
	PUFA	44	1.52 (−19.04, 22.08)	0.882	42	−24.16 (−51.33, 3.01)	0.080
	Carbohydrate	44	−2.15 (−10.43, 6.14)	0.603	42	4.05 (−4.91, 13.02)	0.365
	Sugar	43	−1.93 (−9.31, 5.44)	0.598	42	7.81 (−0.95, 16.58)	0.079
	Fiber	44	−43.64 (−133.44, 46.17)	0.331	42	36.27 (−37.30, 109.85)	0.324
	Protein	44	−9.85 (−27.42, 7.72)	0.263	42	−2.92 (−24.81, 18.97)	0.788
VAT volume, cm^3	Fat	44	−0.29 (−1.88, 1.30)	0.711	42	0.03 (−2.10, 2.15)	0.979
	SFA	44	0.39 (−2.45, 3.22)	0.783	42	0.35 (−3.17, 3.87)	0.841
	MUFA	44	−2.03 (−6.43, 2.37)	0.357	42	2.44 (−3.41, 8.28)	0.403
	PUFA	44	−1.48 (−5.99, 3.02)	0.510	42	−2.80 (−9.02, 3.43)	0.368
	Carbohydrate	44	0.72 (−1.10, 2.53)	0.430	42	0.10 (−1.92, 2.11)	0.924
	Sugar	43	0.42 (−1.26, 2.10)	0.617	42	0.68 (-1.34, 2.70)	0.496
	Fiber	44	−19.93 (−38.84, −1.02)	0.039	42	2.18 (−14.36, 18.71)	0.791
	Protein	44	−2.19 (−6.06, 1.68)	0.258	42	−0.11 (−4.98, 4.75)	0.962

[1]Beta (95% confidence interval (CI)) illustrates the estimated unit change in the respective body composition parameter, associated with an increase of 1% of energy of the macronutrient of interest in the substitution of other macronutrients (isocaloric); regressions were adjusted for sex (except zBMI), pre-pregnancy BMI, gestational age in days, and group allocation. SAT, subcutaneous adipose tissue; VAT, visceral adipose tissue; zBMI, BMI *z* score.

ONLNE SUPPLEMENTAL MATERIAL

SUPPLEMENTAL TABLE 3 Substitution model from late gestation (32nd week of gestation) at birth, 1 year, 3 years, and 5 years[1]

Body Composition Parameter	Macronutrients	Birth [2]			1 year			3 years			5 years		
		n	Beta (95% CI)	*p*	n	Beta (95% CI)	*p*	n	Beta (95% CI)	*p*	n	Beta (95% CI)	*p*
Weight, g	Fat	167	−4.08 (−15.43, 7.27)	0.479	158	−0.70 (−27.83, 26.43)	0.959	150	10.07 (−37.50, 57.65)	0.676	144	13.91 (−56.28, 84.09)	0.696
	SFA	167	1.08 (−17.69, 19.86)	0.910	158	−19.94 (−64.33, 24.45)	0.376	150	−17.84 (96.48, 60.80)	0.655	144	−15.42 (−133.27, 102.42)	0.796
	MUFA	167	−7.28 (−38.35, 23.79)	0.644	158	34.98 (−39.24, 109.20)	0.353	150	50.65 (−79.47, 180.77)	0.443	144	76.89 (−115.51, 269.31)	0.431
	PUFA	167	−25.40 (−58.77, 7.97)	0.135	158	25.57 (−55.02, 106.15)	0.532	150	77.31 (−61.74, 216.35)	0.274	144	80.67 (−128.55, 289.90)	0.447
	Carbohydrate	167	7.24 (−3.33, 17.81)	0.178	158	5.94 (−19.36, 31.24)	0.643	150	−0.21 (−44.57, 44.15)	0.993	144	0.34 (−64.67, 65.35)	0.992
	Sugar	167	9.99 (−0.47, 20.44)	0.061	158	4.60 (−20.46, 29.67)	0.717	150	12.73 (−31.21, 56.67)	0.568	144	5.01 (−58.97 (68.99)	0.877
	Fiber	167	57.66 (−49.82, 165.13)	0.291	158	175.01 (−85.26, 435.27)	0.186	150	420.72 (−29.41, 870.84)	0.067	144	589.93 (−104.18, 1284,04)	0.095
	Protein	167	−18.00 (−44.59, 8.59)	0.183	158	−26.30 (−90.00, 37.38)	0.416	150	−46.34 (−158.71, 66.04)	0.416	144	−64.47 (−232.53, 103.59)	0.449
zBMI[3]	Fat	167	0.00 (−0.02, 0.03)	0.862	158	−0.02 (−0.05, 0.01)	0.179	150	−0.05 (−0.11, 0.01)	0.082	144	−0.00 (−0.02, 0.02)	0.865
	SFA	167	0.01 (−0.03, 0.05)	0.524	158	−0.04 (−0.09, 0.00)	0.054	150	−0.08 (−0.17, 0.02)	0.127	144	−0.01 (−0.05, 0.03)	0.490
	MUFA	167	0.00 (−0.06, 0.07)	0.965	158	−0.01 (−0.09, 0.06)	0.752	150	−0.09 (−0.25, 0.07)	0.259	144	0.02 (−0.05, 0.08)	0.585
	PUFA	167	−0.03 (−0.10, 0.04)	0.405	158	0.00 (−0.08, 0.08)	0.915	150	−0.07 (−0.25, 0.10)	0.404	144	0.01 (−0.06, 0.08)	0.734
	Carbohydrate	167	0.01 (−0.02, 0.03)	0.551	158	0.02 (−0.00, 0.05)	0.060	150	0.06 (0.01, 0.12)	0.023	144	0.01 (−0.01, 0.03)	0.352
	Sugar	167	0.01 (−0.01, 0.04)	0.251	158	0.02 (−0.01, 0.04)	0.121	150	0.07 (0.01, 0.12)	0.015	144	0.01 (−0.01, 0.03)	0.502
	Fiber	167	0.07 (−0.15, 0.30)	0.522	158	0.19 (−0.07, 0.44)	0.155	150	0.34 (−0.22, 0.91)	0.235	144	0.27 (0.05, 0.49)	0.019
	Protein	167	−0.04 (−0.10, 0.01)	0.136	158	−0.05 (−0.11, 0.02)	0.154	150	−0.15 (−0.29, −0.01)	0.037	144	−0.06 (−0.11, −0.00)	0.037
Fat mass, g	Fat	151	−1.70 (−5.64, 2.24)	0.396	153	−3.01 (−14.59, 8.56)	0.608	103	−2.57 (−23.28, 18.13)	0.806	105	−7.32 (−39.84, 25.20)	0.656
	SFA	151	0.38 (−6.02, 6.78)	0.906	153	−9.64 (−28.54, 9.27)	0.315	103	−16.63 (−50.34, 17.09)	0.330	105	−28.04 (−81.38, 25.31)	0.300
	MUFA	151	−4.55 (−15.31, 6.21)	0.405	153	11.47 (−20.47, 43.41)	0.479	103	9.66 (−46.47, 65.78)	0.733	105	8.06 (−80.19, 96.31)	0.857
	PUFA	151	−9.20 (−20.94, 2.54)	0.124	153	−4.62 (−39.35, 30.11)	0.793	103	24.05 (−34.61, 82.70)	0.418	105	24.52 (−65.42, 114.46)	0.590
	Carbohydrate	151	1.65 (−1.98, 5.28)	0.370	153	4.09 (−6.72, 14.89)	0.456	103	5.87 (−12.63, 24.36)	0.530	105	12.42 (−17.07, 41.91)	0.405
	Sugar	151	1.32 (−2.37, 5.01)	0.481	153	3.26 (−7.48, 13.99)	0.550	103	6.52 (−10.97, 24.01)	0.461	105	12,20 (−15.31, 39.70)	0.381
	Fiber	151	20.12 (−18.84, 59.07)	0.309	153	93.09 (−20.39, 206.57)	0.107	103	202.45 (19.28, 385.63)	0.031	105	295.60 (4.52, 586.69)	0.047
	Protein	151	0.53 (−8.44, 9.50)	0.907	153	−5.43 (−32.86, 22.00)	0.696	103	−22.37 (−72.26, 27.53)	0.376	105	−45.16 (−123.00, 32.69)	0.252
Subcutaneous fat area$_{sagittal}$[4], mm^2	Fat	148	−0.30 (−0.64, 0.05)	0.089	146	−0.44 (−0.80, −0.07)	0.020	93	−0.33 (−0.76, 0.10)	0.131	91	−0.35 (−0.81, 0.11)	0.130
	SFA	148	−0.49 (−1.05, 0.08)	0.090	146	−0.74 (−1.34, −0.14)	0.016	93	−0.48 (−1.21, 0.25)	0.191	91	−0.34 (−1.12, 0.44)	0.390
	MUFA	148	−0.32 (−1.24, 0.61)	0.499	146	−0.52 (−1.54, 0.50)	0.314	93	−0.09 (−1.25, 1.07)	0.884	91	−0.37 (−1.57, 0.82)	0.534
	PUFA	148	−0.38 (−1.38, 0.61)	0.446	146	−0.37 (−1.49, 0.74)	0.508	93	−0.84 (−2.01, 0.34)	0.161	91	−1.05 (−2.31, 0.21)	0.100
	Carbohydrate	148	0.24 (−0.08, 0.56)	0.143	146	0.45 (0.11, 0.79)	0.010	93	0.31 (−0.07, 0.70)	0.106	91	0.36 (−0.04, 0.77)	0.078
	Sugar	148	0.16 (−0.16, 0.47)	0.338	146	0.20 (−0.14, 0.54)	0.248	93	0.38 (0.03, 0.72)	0.032	91	0.41 (0.04, 0.77)	0.031
	Fiber	148	1.91 (−1.39, 5.21)	0.255	146	4.22 (0.64, 7.80)	0.021	93	3.07 (−0.74, 6.89)	0.113	91	1.75 (−2.32, 5.82)	0.395
	Protein	148	0.03 (−0.76, 0.81)	0.948	146	−0.40 (−1.32, 0.52)	0.396	93	−0.18 (−1.12, 0.74)	0.694	91	−0.70 (−1.75, 0.35)	0.191
Preperitoneal fat area$_{sagittal}$[4], mm^2	Fat	141	0.01 (−0.10, 0.11)	0.920	145	0.00 (−0.17, 0.17)	0.990	92	0.05 (−0.38, 0.49)	0.813	90	−0.23 (−0.77, 0.30)	0.385
	SFA	141	0.01 (−0.16, 0.19)	0.874	145	−0.19 (−0.46, 0.09)	0.185	92	0.12 (−0.62, 0.85)	0.754	90	−0.43 (−1.33, 0.48)	0.349
	MUFA	141	0.04 (−0.25, 0.32)	0.804	145	0.23 (−0.23, 0.69)	0.325	92	0.10 (−1.06, 1.26)	0.861	90	−0.60 (−1.98, 0.79)	0.393
	PUFA	141	−0.06 (−0.37, 0.25)	0.697	145	0.35 (−0.16, 0.85)	0.175	92	−0.10 (−1.28, 1.07)	0.861	90	0.05 (−1.42, 1.53)	0.944
	Carbohydrate	141	−0.03 (−0.13, 0.07)	0.610	145	−0.04 (−0.19, 0.12)	0.667	92	−0.01 (−0.40, 0.38)	0.946	90	0.19 (−0.29, 0.67)	0.427
	Sugar	141	−0.02 (−0.12, 0.08)	0.694	145	0.00 (−0.15, 0.16)	0.988	92	0.19 (−0.16, 0.54)	0.286	90	0.34 (0.09, 0.77)	0.122
	Fiber	141	0.211 (−0.81, 1.23)	0.682	145	0.28 (−1.37, 1.93)	0.736	92	−0.461 (−4.33, 3.41)	0.814	90	1.21 (−3.51, 5.94)	0.611
	Protein	141	0.05 (−0.18, 0.29)	0.650	145	0.19 (−0.22, 0.61)	0.362	92	−0.22 (−1.15, 0.70)	0.635	90	−0.37 (−1.61, 0.87)	0.553

[1]Beta (95% confidence interval (CI)) illustrates the estimated unit change in the respective body composition parameter, associated with an increase of 1% of energy of the macronutrient of interest in the substitution of other macronutrients (isocaloric); regressions were adjusted for sex (except zBMI), pre-pregnancy BMI, gestational age in days, and group allocation; z BMI, BMI *z* score.[2] Ultrasound measurements were performed at 6 wk postpartum.[3] zBMI according to (1). [4] Subcutaneous and preperitoneal fat were measured as areas of 1-cm length in sagittal plane in the middle of the xiphoid process according to Brei et al. (2).

ONLNE SUPPLEMENTAL MATERIAL

REFERENCES

1. Kromeyer-Hauschild K, Wabitsch M, Kunze D, Geller F, Geiß HC, Hesse V, Hippel von A, Jaeger U, Johnsen D, Korte W, et al. Percentiles of body mass index in children and adolescents evaluated from different regional German studies. Monatsschrift Kinderheilkunde 2001;8(149):807–19.

2. Brei C, Much D, Heimberg E, Schulte V, Brunner S, Stecher L, Vollhardt C, Bauer JS, Amann-Gassner U, Hauner H. Sonographic assessment of abdominal fat distribution during the first year of infancy. Pediatr Res 2015;78(3):342–50. doi: 10.1038/pr.2015.108.
